# Clinical significance of positron emission tomography-computed tomography in the classification of thymic tumors

**DOI:** 10.1093/icvts/ivaf065

**Published:** 2025-03-12

**Authors:** Jian Gao, Si-Yang Wang, Yong-Qiang Ao, Jia-Hao Jiang, Miao Lin, Shuai Wang, Hong-Cheng Shi, Jian-Yong Ding

**Affiliations:** Department of Thoracic Surgery, Zhongshan Hospital, Fudan University, Shanghai, China; Cancer Center, Zhongshan Hospital, Fudan University, Shanghai, China; Cancer Center, Zhongshan Hospital, Fudan University, Shanghai, China; Department of Nuclear Medicine, Zhongshan Hospital, Fudan University, Shanghai, China; Department of Thoracic Surgery, Zhongshan Hospital, Fudan University, Shanghai, China; Cancer Center, Zhongshan Hospital, Fudan University, Shanghai, China; Department of Thoracic Surgery, Zhongshan Hospital, Fudan University, Shanghai, China; Cancer Center, Zhongshan Hospital, Fudan University, Shanghai, China; Department of Thoracic Surgery, Zhongshan Hospital, Fudan University, Shanghai, China; Cancer Center, Zhongshan Hospital, Fudan University, Shanghai, China; Department of Thoracic Surgery, Zhongshan Hospital, Fudan University, Shanghai, China; Cancer Center, Zhongshan Hospital, Fudan University, Shanghai, China; Cancer Center, Zhongshan Hospital, Fudan University, Shanghai, China; Department of Nuclear Medicine, Zhongshan Hospital, Fudan University, Shanghai, China; Department of Thoracic Surgery, Zhongshan Hospital, Fudan University, Shanghai, China; Cancer Center, Zhongshan Hospital, Fudan University, Shanghai, China

**Keywords:** thymic tumours, PET-CT, SUVmax, lymph node metastasis

## Abstract

**OBJECTIVES:**

This study aimed to explore the possibility of positron emission tomography/computed tomography (PET-CT) in identifying histological classification of thymic tumours.

**METHODS:**

Patients diagnosed as thymic tumours and accepted PET-CT scans were included. Thymic tumours were classified into three subgroups: low-risk thymoma (A, AB and B1), high-risk thymoma (B2, B3) and thymic carcinoma (TC). Logistic regression analysis was performed to identify potential factors differentiating the classification of thymic tumours. The receiver operating characteristic curve was applied to assess the diagnosis efficiency and the cut-off value.

**RESULTS:**

From 2015 to 2023, a total of 176 patients, including 75 cases of low-risk thymoma, 60 cases of high-risk thymoma and 41 cases of TC, were included. The logistic regression models suggested maximum standardized uptake value (SUVmax) as a potential factor differentiating the three subgroups. Moreover, the receiver operating characteristic curve identified the SUVmax in differentiating low-risk thymoma vs high-risk thymoma (area under the curve [AUC]: 0.845, 95% CI: 0.776–0.913, specificity: 0.907, sensitivity: 0.716), low-risk thymoma vs TC (AUC: 0.976, 95% CI: 0.953–0.999, specificity: 0.933, sensitivity: 0.951) and high-risk thymoma vs TC (AUC: 0.84, 95% CI: 0.761–0.92, specificity: 0.865, sensitivity: 0.703), respectively. SUVmax was also an independent factor identifying thymic tumours with or without lymph node metastasis. The cut-off of 10 in SUVmax could well identify lymph node metastasis with the positive predict value of 0.684 and negative predict value of 0.981.

**CONCLUSIONS:**

SUVmax is a reliable factor in distinguishing different histological subgroups and identifying lymph node metastasis in thymic tumours.

## GRAPHICAL ABSTRACT

**Figure:**
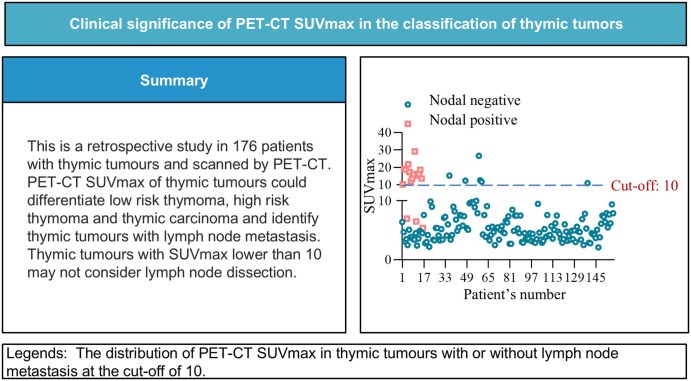

## INTRODUCTION

Thymic epithelial tumours (TETs), including thymoma and thymic carcinoma (TC), are relatively rare thoracic neoplasms with an incidence of approximately 1.5–3.2/million per year [1]. According to the World Health Organization (WHO) classification, thymic tumours could be divided into A, AB, B1, B2, B3 subtypes and TC. Accumulated studies suggested that histological subtypes could affect the prognosis and further divided thymoma into low-risk thymoma (A, AB and B1) and high-risk thymoma (B2 and B3) [2, 3]. Compared to the low-risk thymoma, the high-risk thymoma is more likely to have local invasion and may require multimodal therapy [4].

Compared with the conventional Masaoka staging system, the latest eighth TNM staging system (tumor stage evaluation system) emphasized the systemic evaluation of lymph nodes, including N1, located in the anterior mediastinum, and N2, in the deep mediastinum [5]. Because TETs are rare, the clinical patterns and features of lymph node metastasis remain elusive, leading to difficulty in the choice for intraoperative lymph node dissection. A multicentre retrospective study found that no nodal involvement was found in low-risk thymoma. The incidences of nodal metastasis in high-risk thymoma (B2/B3) and TC were 1.3% and 7.9%, respectively [6]. Another multicentre prospective observational study of lymph node metastasis also identified high-risk patients with aggressive histology and advanced T category [7]. However, these predictive models are mainly based on postoperative pathology classification. The latest National Comprehensive Cancer Network (NCCN) guidelines do not recommend biopsy for resectable TETs. Thus, a noninvasive method for predicting lymph node metastasis in TETs needs to be identified.

Fluoro-2-deoxy-D-glucose positron emission tomography/computed tomography (^18^F-FDG PET-CT) has advantages in evaluating thymic tumours, including stage prediction and differential diagnosis [8]. However, it also has limitations in the direct evaluation of mediastinal lymph nodes, especially in nodes with diameters less than 1 cm and N1 lymph nodes. A retrospective study based on 56 patients with TETs reported that the maximum Standardized uptake value (SUVmax) of tumours in PET-CT could differentiate histological classification and the Masaoka-Koga stage [9]. Since lymph node metastasis mostly occurred in TETs of higher histological grade, we suspected that metabolic parameter (SUVmax) of the tumours in PET-CT may also contribute to the prediction of lymph node metastasis of TETs. Thus, this study mainly aimed to explore the feasibility of PET-CT in the classification of thymic tumours.

## PATIENTS AND METHODS

### Sample selection

The protocol of this retrospective, single-centre study was reviewed and approved by the ethics committee of Zhongshan Hospital Fudan University (Y2023-078, 2/23/2023). The written informed consent has been obtained from all patients included in this study. Any collection and storage of data or biological material from research participants for multiple and indefinite use should be consistent with requirements outlined in the WMA Declaration of Taipei. The inclusion criteria were as follows: (1) Patients were diagnosed with thymoma, TC and neuroendocrine thymic tumour (NETT, classified as TC) based on pathology. (2) All patients underwent PET-CT before thymectomy. (3) Patients who accepted either minimally invasive or open thymectomy with the dissection of either N1 or N2 lymph nodes were acceptable. The pathology of the dissected tumours and lymph nodes was verified by two experienced pathologists. The exclusion criteria were as follows: (1) The TETs were unresectable (patients with thymic tumours suspected to have distant metastasis (brain, liver, etc.), invasion into the aorta, myocardium or widely dissemination in the chest cavity). (2) Patients had accepted neoadjuvant therapy (chemotherapy or radiotherapy) before PET-CT, since neoadjuvant therapy might influence the image and metabolism parameters of the tumour. (3) Neither the N1 nor the N2 lymph nodes were dissected during surgery. (4) Patients had undergone thymectomy before and then relapsed. (5) Mediastinal tumours including germ cell tumours, lymphoma, benign lesions and metastatic thyroid carcinoma. Based on these criteria, a total of 176 patients from 2011 to 2023 were included in this study.

### Data collection

The data presented in this study included the basic demographic indices: age, sex and myasthenia gravis (MG). The following image and metabolic parameters of the PET-CT were included in this study: tumour size, local invasion, CT value and SUVmax. MG was diagnosed by an experienced neurologist (Ji-Hong Dong) based on MG symptoms, blood examination and electromyography. Local invasion referred to tumours invading the pleura, pericardium, lung, superior vena cava and innominate vein. Tumour histology was classified according to the WHO classification system [10].

### PET-CT protocol and evaluation

All patients were scanned by ^18^F-FDG PET/CT with a standard PET-CT protocol at this institute [11]. Before the examination, patients were required to fast for approximately 6 hours and measure the serum glucose level, which should be less than 180 mg/dl. Then, they were intravenously injected with 370–555 MBq (10–15 mCi) of ^18^F-FDG based on their weight and remained in a seated position during the uptake period lasting approximately 60 min. A PET-CT device (Discovery VCT unit, GE Medical Systems, Waukesha, USA) was then used to scan from the head to the upper thighs. The PET-CT images and metabolic conditions were displayed and evaluated in the uWS-MI R001 workstation (United Imaging, Shanghai, China). The images were independently evaluated by two nuclear medicine physicians (Hong-Cheng Shi and Si-Yang Wang) who were blinded to the pathology of these patients. The SUVmax of the tumour was calculated using the attenuation-corrected images, which could adjust for the injected FDG dose, patient weight and cross-calibration factors between the PET and the dose calibrator. The estimated threshold was 42% of the maximum value.

### Statistical analysis

Statistical analysis was performed with IBM SPSS 23.0 (SPSS, Chicago, IL) or R 3.5.1 statistical software (http://www.r-project.org). Categorical variables were analysed using the chi-squared test. Continuous variables were compared via Student’s *t* test. Categorical variables with low frequencies were compared by Fisher’s exact test and continuous variables with non-normal distribution was compared with Mann–Whitney *U* test. For comparison of three groups, one-way analysis of variance (ANOVA) test was performed. To identify potential independent factors predicting nodal involvement, univariate binary logistic regression was performed, followed by a multivariable analysis including only variables with *P* < 0.05 in the univariate analysis. The multicollinearity of the included factors was verified using VIF. The predictive value and the cut-off were verified by receiver operating characteristic (ROC) curve analysis. The maximum of Youden index (sensitivity and 1-specificity) was used to identify the optimal cut-off. *P* < 0.05 was regarded as significance. Missing data were completed using the multiple imputation with the mice package [12].

## RESULTS

**Figure 1:**
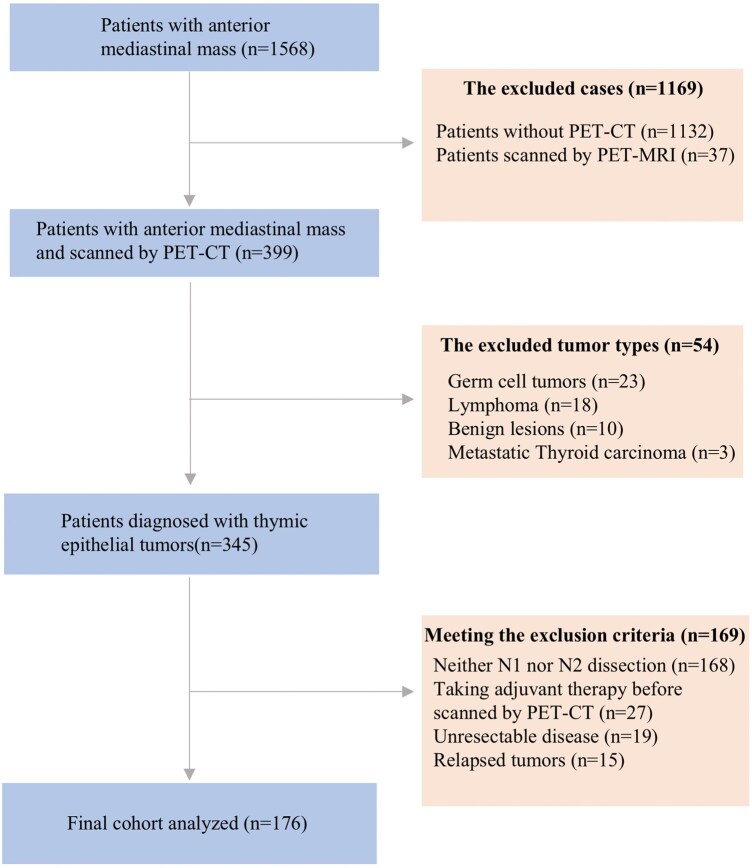
Diagram of the patient selection process. Initially, a total of 399 patients diagnosed with anterior mediastinal masses and scanned by PET-CT were identified. After the exclusion process, a total of 156 patients were finally included in the study.

From 2015 to 2023, a total of 399 patients with anterior mediastinal mass and PET-CT were identified. Based on the exclusion criteria, a total of 176 patients were finally included in the study (Fig. 1). The clinical histopathological characteristics of the cohort are presented in Table 1. This cohort contained 91 males and 85 females with a mean age of 52.82 ± 11.8 years. Only 12 patients were identified to have MG symptoms. In terms of the pathology classification, 75 patients were classified as the low-risk group, 60 patients were identified as the high-risk group and 41 patients had TC. Local invasion judged from PET-CT and contrast-enhanced chest CT was found in 87 patients. Finally, a total of 16 patients were identified to have lymph node metastasis (9.09%) based on pathological results.

**Table 1:** The clinical and histological profiles of patients with TETs

Variables	Patients
Gender	
Male	91
Female	85
Age (years)	51.82 ± 11.8
Tumour size (cm)	5.14 ± 2.36
MG	
No	164
Yes	12
Pathology	
Low-risk thymoma	75
High-risk thymoma	60
TC	41
CT value	39.56 ± 8.78
SUVmax	6.43 ± 4.26
Local invasion	
No	89
Yes	87
Lymph node metastasis	
No	160
Yes	16

**Figure 2:**
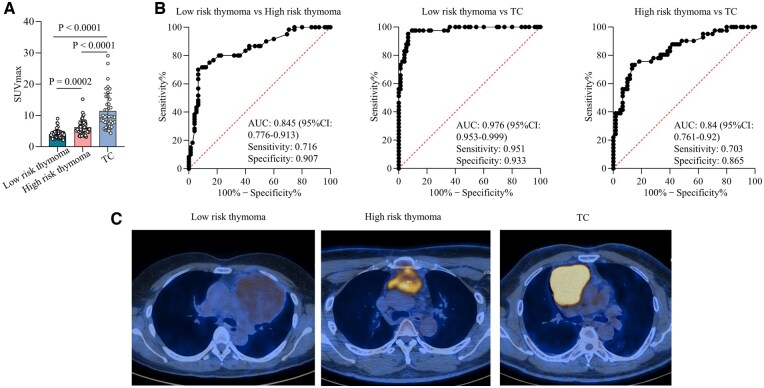
(**A**) The SUVmax in the three subgroups. One-way ANOVA test was performed; (**B**) ROC analysis on SUVmax predicting the histological classification of thymic tumours (low-risk thymoma, high-risk thymoma and TC); (**C**) representative PET-CT images of low-risk thymoma, high-risk thymoma and TC.

Next, we analysed the difference among patients with low-risk thymoma, high-risk thymoma and TC, and the results are presented in Table 2. The age of patients with TC was obviously higher than patients with thymoma (56.02 ± 10.26 vs 51.12 ± 11.31 in low-risk thymoma and 49.82 ± 12.91 in high-risk thymoma, *P* = 0.027), and the SUVmax was gradually increased from low-risk thymoma (3.95 ± 1.22), high-risk thymoma (6.15 ± 2.16) to TC (11.36 ± 5.77, *P* < 0.001) (Fig. 2A and Table 2). Moreover, the multivariable logistic regression analyses were performed to identify factors differentiating different histological subgroups in three models (model 1: low-risk thymoma vs high-risk thymoma; model 2: low-risk thymoma vs TC; model 3: high-risk thymoma vs TC). It presented that SUVmax was an independent factor to differentiate the three groups in all models with the odds ratio (OR, 95% CI) of 2.504 (1.792–3.498) in model 1, 3.478 (2.157–5.701) in model 2 and 1.589 (1.289–1.958) in model 3 (Table 3).

**Table 2:** The comparison of patients in different histological subgroups

Variables	Low-risk thymoma	High-risk thymoma	TC	*P*
	*N* = 75	*N* = 60	*N* = 41	
Gender				0.108
Male	32	34	25	
Female	43	26	16	
Age (years)	51.12 ± 11.31	49.82 ± 12.91	56.02 ± 10.26	0.027
Tumour size(cm)	5.08 ± 2.39	5.45 ± 2.44	4.81 ± 2.17	0.391
MG				0.127
No	69	54	41	
Yes	6	6	0	
CT value	39.91 ± 9.2	39.43 ± 8.14	39.12 ± 9.09	0.892
SUVmax	3.95 ± 1.22	6.15 ± 2.16	11.36 ± 5.77	<0.001
Local invasion				
No	42	28	19	0.462
Yes	33	32	22	

**Tables 3:** Multivariable logistic regression analysis on factors differentiating different histological subgroups

Variables	OR (95% CI)	*P*	Tolerance	VIF
Model 1: low-risk thymoma vs high-risk thymoma				
Univariate analysis				
Gender (male vs female)	0.569 (0.287–1.13)	0.087	0.949	1.054
Age	0.991 (0.963–1.019)	0.53	0.965	1.036
Tumour size	1.065 (0.924–1.226)	0.385	0.671	1.49
Local invasion (Yes vs No）	1.455 (0.735–2.877)	0.282	0.718	1.392
MG symptom (Yes vs No)	1.278 (0.39–4.185)	0.685	0.883	1.132
CT value	0.994 (0.956–1.033)	0.749	0.924	1.082
SUVmax	2.504 (1.792–3.498)	<0.001	0.886	1.128
Multivariate analysis				
SUVmax	2.504 (1.792–3.498)	<0.001		
Model 2: low-risk thymoma vs thymic carcinoma				
Univariate analysis				
Gender (male vs female)	0.476 (0.219–1.035)	0.061	0.872	1.147
Age	1.043 (1.005–1.083)	0.026	0.928	1.077
Tumour size	0.947 (0.8–1.122)	0.947	0.751	1.331
Local invasion (Yes vs No）	1.474 (0.686–3.166)	0.32	0.752	1.329
CT value	0.991 (0.95–1.033)	0.658	0.913	1.095
SUVmax	3.507 (2.157–5.701)	<0.001	0.86	1.163
Multivariate analysis				
SUVmax	3.478 (2.157–5.701)	<0.001		
Model 3: high-risk thymoma vs thymic carcinoma				
Univariate analysis				
Gender (male vs female)	0.837 (0.373–1.879)	0.666	0.895	1.117
Age	1.046 (1.009–1.085)	0.015	0.876	1.142
Tumour size	0.881 (0.732–1.061)	0.181	0.747	1.338
Local invasion (Yes vs No）	1.013 (0.457–2.246)	0.974	0.736	1.36
CT value	0.996 (0.95–1.044)	0.861	0.948	1.055
SUVmax	1.592 (1.284–1.973)	<0.001	0.886	1.129
Multivariate analysis				
SUVmax	1.589 (1.289–1.958)	<0.001		

ROC analyses were performed to evaluate the efficiency of SUVmax distinguishing the three histological subgroups (Fig. 2B). The area under the curve (AUC) values were 0.845 (95% CI: 0.776–0.913), 0.976 (95% CI: 0.953–0.999) and 0.84 (95% CI: 0.761–0.92), respectively, in low-risk thymoma vs high-risk thymoma, low-risk thymoma vs TC and high-risk thymoma vs TC. The representative PET- CT images showing metabolic status of the three subgroups are presented in Fig. 2C.

**Figure 3:**
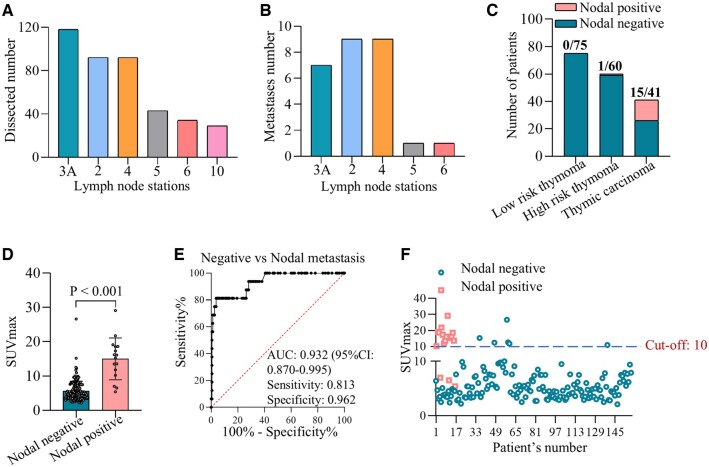
(**A**) The dissection profiles of lymph nodes during thymectomy; (**B**) the distribution of metastatic lymph nodes in 16 patients with TETs; (**C**) the incidence of lymph node metastasis in low-risk thymoma, high-risk thymoma and TC; (**D**) the difference of SUVmax in TETs with or without lymph node metastasis; (**E**) ROC analysis on SUVmax predicting lymph node metastasis in TETs; (**F**) the distribution of SUVmax in TETs with or without lymph node metastasis at the cut-off of 10.

We further analysed the difference of TETs with or without lymph node metastasis. The dissection profiles of lymph nodes were provided. The dissected lymph nodes including station 3a (*n* = 118), 2 (*n* = 92), 4 (*n* = 92), 5 (*n* = 43), 6 (*n* = 34) and 10 (*n* = 29) (Fig. 3A). The most common metastatic lymph nodes were station 2 (*n* = 9), 4 (*n* = 9) followed by 3a (*n* = 7), 5 (*n* = 1) and 6 (*n* = 1) (Fig. 3B). Among the 16 patients with lymph node metastasis, 15 patients were diagnosed as TC and only 1 patient with high-risk thymoma had lymph node metastasis (Fig. 3C). Univariate and multivariate logistic regression analyses were used to determine the independent association and discriminatory accuracy of potential variables in identifying patients with lymph node metastasis (Table 4). It presented that SUVmax of TET was an independent factor to predict lymph node metastasis with the OR (95% CI) of 1.509 (1.286–1.77) (*P* < 0.001). The SUVmax of TETs with lymph node metastasis was obviously higher than those with no metastasis (Fig. 3D). Then, the efficiency of SUVmax distinguishing TETs with or without lymph node metastasis was evaluated via the ROC analysis. The AUC was 0.932 (95% CI: 0.87–0.995) with the sensitivity of 0.813 and specificity of 0.962 (Fig. 3E). The cut-off of SUVmax determined by Youden index was 10 with the positive predictive value (PPV) of 0.684 (95% CI: 0.491–0.874) and negative predictive value (NPV) of 0.981 (95% CI: 0.945–0.996) (Supplementary Table S1 and Fig. 3F). The representative PET-CT images of thymic tumours with or without nodal metastases are presented in Supplementary Fig. S1.

**Table 4:** Multivariable logistic regression analysis on factors predicting lymph node metastasis in TETs

Variables	OR (95% CI)	*P*	Tolerance	VIF
Univariate analysis				
Gender (male vs female)	0.22 (0.06–0.8)	0.022	0.915	1.092
Age	1.056 (1.004–1.111)	0.035	0.929	1.077
Tumour size	1.107 (0.907–1.35)	0.317	0.711	1.406
Local invasion (Yes vs No)	3.4 (1.052–10.992)	0.041	0.72	1.389
MG symptom (Yes vs No)	0.903 (0.109–7.485)	0.925	0.9	1.112
CT value	0.958 (0.907–1.011)	0.12	0.93	1.076
SUVmax	1.509 (1.286–1.77)	<0.001	0.883	1.112
Multivariate analysis				
SUVmax	1.509 (1.286–1.77)	<0.001		

## DISCUSSION

In this work, we performed a retrospective study in 176 patients diagnosed as TETs and scanned by PET-CT to explore the clinical significance of PET-CT SUVmax in the classification of TETs. The results ultimately presented that SUVmax could distinguish low-risk thymoma, high-risk thymoma and TC and identify TETs with lymph node metastasis, which provided an effective instruction for the thymectomy.

Previously, many studies have discussed the potential value of PET-CT in the diagnosis, staging, grading of malignancies and evaluation of the pathological response to adjuvant therapy for malignant TETs [13–15]. SUVmax in PET-CT represents the highest glucose uptake in tumour tissues and is widely applied in clinical practice. Several studies have reported the high accuracy of PET-CT in discriminating thymoma from TC. Han *et al.* [16] demonstrated that SUVmax had excellent ability to differentiate TC from low-risk/high-risk thymoma. Another study also observed the increased uptake of FDG in the order of low-risk thymomas to high-risk thymomas to TCs [17]. In this study based on 176 patients diagnosed as TETs and scanned by PET-CT, we confirmed that PET-CT SUVmax could serve as an independent factor to differentiate the three subgroups. The prediction efficiency of SUVmax in low-risk thymoma vs high-risk thymoma was poorer than low-risk thymoma vs TC but similar with high-risk thymoma vs TC. Thus, it was best performed in low-risk thymoma vs TC. The classification of thymic tumours was mainly based on the proportion of lymphocytes and malignant thymic epithelial cells. Also, the components of high-risk thymoma especially B3 thymoma is close to TC, which might lead to the overlap in SUVmax between high-risk thymomas and TC.

Accurate preoperative evaluations of nodal metastasis are limited and difficult for thoracic surgeons, and lymph node dissection is still not regarded as a routine procedure during thymectomy, which might be attributed to the low incidence of lymph node metastasis in TETs. A Japanese database containing 1320 patients with thymic tumours reported that the overall incidence of lymph node metastasis was 5.9%. The incidences of lymph node involvement in patients with thymoma and TC were 1.8% and 27%, respectively [18]. Another multicentre study found that the overall rate of lymph node metastasis was approximately 5.5%, with 2.1% in thymoma, 25% in TCs and 50% in NETT. The prognosis of patients with lymph node involvement was much worse than that of patients without lymph node involvement [6]. Collectively, these multicentre studies revealed that lymph node metastasis is rare in thymoma and relatively common in TC. In this study, the overall incidence of lymph node metastasis was approximately 9.09% (16/176), with 0.74% (1/135) in patients with thymoma and 36.5% (15/41) in patients with TC (including seven NETTs). The relatively higher incidence of lymph node metastasis in this study could be attributed to the following reasons. First, the proportion of TC in this study was approximately 23.3% (41/176), and most cases with lymph node metastasis were TC. Second, all patients in this study underwent PET-CT for further evaluation. In clinical practice, PET-CT is usually provided for patients with advanced-stage tumours.

Considering the large disparity between thymoma and thymic tumours in the incidence of lymph node metastasis, we hypothesized that the imaging and metabolic parameters of PET-CT could predict lymph node metastasis in thymic tumours. In this study, the results showed that SUVmax was obviously higher in TETs with lymph node involvement. Logistic regression analysis identified SUVmax as an independent factor predicting lymph node metastasis. The ROC analysis showed high accuracy, with AUC of 0.932 (specificity: 0.962, sensitivity: 0.813). The relatively low PPV (0.684, 95% CI: 0.526–0.874) might be attributed to the small number of patients with lymph node metastasis (*N* = 16). In contrast, the high NPV (0.978, 95% CI: 0.937–0.995) could identify most patients without lymph node metastasis. In this study, we found that most cases of lymph node metastases happened in station 3a (*n* = 7), 2 (*n* = 9) and 4 (*n* = 9). Thus, the three stations might be considered for dissection when SUVmax higher than 10. Because of the high expense of PET-CT, thymic tumours considered to have local advance of potentially distant metastasis were advised to have PET-CT scan, which restrained the boarder application of PET-CT. In addition, we also noticed two NETTs (belonging to TC) with SUVmax <10 and lymph node metastasis in this study. Thus, lymph node dissection might still be necessary in for NETTs with SUVmax <10. While the eighth TNM staging system emphasize the evaluation of N1 and N2 lymph nodes for thymic tumours, the surgical guideline on lymph node dissection is still absent. In our institution, N1 lymph nodes are included in the scope of routine total thymectomy, N2 lymph nodes are only performed in high-risk tumours such as neuroendocrine tumours or patients with lymph node enlargement on imaging, and N2 is not routinely cleared during thymectomy. In consistent with other study, this study confirmed that PET-CT SUVmax could distinguish different histology types (low-risk thymoma, high-risk thymoma and TC). However, our study first reported that the SUVmax of thymic tumours could predict the lymph node metastasis and found that thymic tumours with SUVmax >10 had obviously higher risk for nodal metastasis. Thus, this point added novel insight beyond those already established in the literature for many years. In this study, most cases with nodal metastasis were diagnosed as TC, and only one patient with high-risk thymoma (SUVmax = 11.2) was identified to have nodal metastasis. In low-risk thymoma and high-risk thymoma groups, the SUVmax of most cases was also lower than 10 (136/139), which led to the situation that our data concerning the risk of nodal metastases mainly refer to patients with TC. While the rate of nodal metastasis in thymoma (approximate 1%) is much lower than that in TC, thymoma with SUVmax higher than 10 should also consider the nodal dissection.

This study was also subject to some limitations. First, this study was retrospective, which could lead to selection bias. Second, only 16 patients in this study were identified to have lymph node metastasis. Most cases of nodal metastasis happened in TC (15/41, 36.5%), and only one patient diagnosed as thymoma had lymph node metastasis (1/135, 0.74%). Thus, the relative low incidence of lymph node metastasis in thymoma led to the limited sample size. The retrospective nature and small sample size of patients with lymph node metastasis in this study might potentially influence the cut-off of SUVmax differentiating thymic tumours with or without lymph node metastasis in broader clinical settings and need further verification in multicentre studies. Thirdly, while we discovered that SUVmax of TETs could predict lymph node metastasis, we did not determine the exact lymph node that should be dissected due to the limited number of patients. Finally, PET-CT is expensive and not a routine examination for every anterior mediastinal mass in clinical practice. In this study, a total of 75% cases (12/16) of thymic tumours with lymph node metastasis had local invasion. Thus, we recommended PET-CT at least in patients with locally advanced thymic tumours.

## CONCLUSIONS

In conclusion, this study confirmed the feasibility of PET-CT SUVmax in distinguishing different histological subgroups and predicting lymph node metastasis in TETs, which provided a noninvasive method to instruct lymph node dissection in thymectomy.

## Supplementary Material

ivaf065_Supplementary_Data

## Data Availability

The datasets used and/or analysed during the current study are available from the corresponding author on reasonable request. **Jian Gao:** Conceptualization; Data curation; Formal analysis; Methodology; Writing—original draft. **Si-Yang Wang:** Data curation; Software; Writing—review & editing. **Yong-Qiang Ao:** Formal analysis; Methodology. **Jia-Hao Jiang:** Data curation. **Miao Lin:** Methodology; Visualization. **Shuai Wang:** Data curation; Formal analysis. **Hong-Cheng Shi:** Conceptualization; Writing—review & editing. **Jianyong Ding:** Conceptualization; Supervision; Writing—review & editing Interdisciplinary CardioVascular and Thoracic Surgery thanks Larry R. Kaiser and the other anonymous reviewers for their contribution to the peer review process of this article.

## Notes

Presented at the 32nd European Conference on General Thoracic Surgery, Barcelona, Spain, May 28, 2024.

## ABBREVIATIONS

AUC: Area under the curve
18F-FDG: Fluoro-2-deoxy-D-glucose
MG: Myasthenia gravis
NPV: Negative predictive value
PET-CT: Positron emission tomography/computed tomography
PPV: Positive predictive value
ROC: Receiver operating characteristic
SUVmax: Maximum Standardized uptake value
TETs: Thymic epithelial tumours
WHO: World Health Organization
