# Supplementary material for: Clinical significance of positron emission tomography-computed tomography in the classification of thymic tumors
Source: Interdiscip Cardiovasc Thorac Surg. 2025 Mar 12;40(3):ivaf065. doi: 10.1093/icvts/ivaf065 (PMC11928932; doi:10.1093/icvts/ivaf065)
Supplement: ivaf065_Supplementary_Data [file ivaf065_supplementary_data.zip › CORRECT Supplementary figure legends.docx]

**Supplementary figure 1:** Representative PET-CT images of TETs with or without nodal metastasis.
