# Supplementary material for: Clinical significance of positron emission tomography-computed tomography in the classification of thymic tumors
Source: Interdiscip Cardiovasc Thorac Surg. 2025 Mar 12;40(3):ivaf065. doi: 10.1093/icvts/ivaf065 (PMC11928932; doi:10.1093/icvts/ivaf065)
Supplement: ivaf065_Supplementary_Data [file ivaf065_supplementary_data.zip › CORRECT Supplementary tables.docx]

| SUVmax Cutoff = 10 | Nodal positive | Nodal negative | PPV (95%CI) | NPV (95%CI) |
| --- | --- | --- | --- | --- |
| PET-CT positive | 13 | 6 | 0.684 | 0.981 |
| PET-CT negative | 3 | 154 | 0.491-0.874 | 0.945-0.996 |

Supplementary table 1: ROC analysis on the threshold of SUVmax differentiating TETs with or without lymph node metastasis

Note: PPV, positive predict value; NPV, negative predict value.
