# Supplementary figures and images for: Clinical significance of positron emission tomography-computed tomography in the classification of thymic tumors
Source: Interdiscip Cardiovasc Thorac Surg. 2025 Mar 12;40(3):ivaf065. doi: 10.1093/icvts/ivaf065 (PMC11928932; doi:10.1093/icvts/ivaf065)

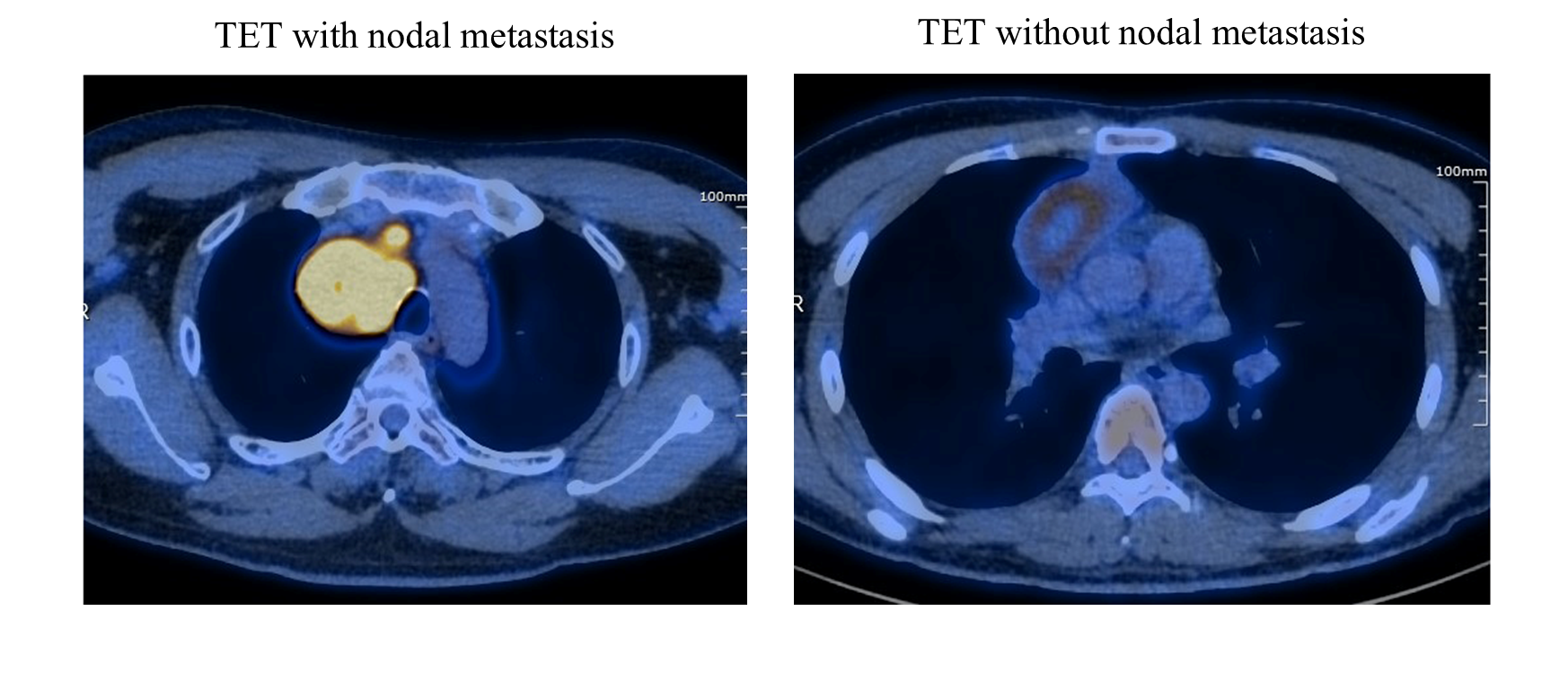

Supplement: ivaf065_Supplementary_Data [file ivaf065_supplementary_data.zip › Supplementary figure 1.tif]
